# Double Entropy Joint Distribution Function and Its Application in Calculation of Design Wave Height

**DOI:** 10.3390/e21010064

**Published:** 2019-01-14

**Authors:** Guilin Liu, Baiyu Chen, Song Jiang, Hanliang Fu, Liping Wang, Wei Jiang

**Affiliations:** 1 College of Engineering, Ocean University of China, Qingdao 266100, China; 2 College of Engineering, University of California Berkeley, Berkeley, CA 94720, USA; 3 School of Management, Xi’an University of Architecture and Technology, Xi’an 710055, China; 4 School of Mathematical Sciences, Ocean University of China, Qingdao 266100, China; 5 School of Forest Resources and Conservation, University of Florida, Gainesville, FL 32611, USA

**Keywords:** maximum entropy function, copula function, recurrence level

## Abstract

Wave height and wave period are important oceanic environmental factors that are used to describe the randomness of a wave. Within the field of ocean engineering, the calculation of design wave height is of great significance. In this paper, a periodic maximum entropy distribution function with four undetermined parameters is derived by means of coordinate transformation and solving conditional variational problems. A double entropy joint distribution function of wave height and wave period is also derived. The function is derived from the maximum entropy wave height function and the maximum entropy periodic function, with the help of structures of the Copula function. The double entropy joint distribution function of wave height and wave period is not limited by weak nonlinearity, nor by normal stochastic process and narrow spectrum. Besides, it can fit the observed data more carefully and be more widely applicable to nonlinear waves in various cases, owing to the many undetermined parameters it contains. The engineering cases show that the recurrence level derived from the double entropy joint distribution function is higher than that from the extreme value distribution using the single variables of wave height or wave period. It is also higher than that from the traditional joint distribution function of wave height and wave period.

## 1. Introduction

Longuet-Higgins [1] deduced the distribution function of wave elements for the first time under the assumption that wave surface displacement is a stationary normal stochastic process and under the narrow spectrum. The conclusion is that the distribution of wave elements is Rayleigh. Due to the backwardness of early ocean observation techniques and means, the observation data were insufficient and were inaccurate, making it impossible to accurately judge whether actual wave elements were in good agreement with this distribution. With the development of ocean observation technology, the measured data and laboratory data proved that the Rayleigh distribution of wave elements is reasonable under the assumption that the wave surface displacement is a normal stochastic process. Such distribution can also well describe some problems (e.g., deep-water waves), but its application is greatly limited because the derivation of this distribution is based on the assumption that the waves are normal stochastic processes. For example, when studying problems of ocean microwave remote sensing and modern ocean military technology, we need to study abnormal waves [2,3,4].

In recent years, the study of wave element distribution function has been deepening, but most of the deriving results are still based on the assumption that the wave surface displacement is a normal stochastic process, and brought the same result that distribution of wave elements is Rayleigh. Although this assumption greatly simplifies the theoretical analysis and derivation, however, in fact, not all wave elements follows the Rayleigh distribution [5,6]. In 1954, a Soviet scholar deduced a distribution of wave period under the condition that the wavelength conforms to the Rayleigh distribution. This distribution is in good agreement with some actual situations, but it cannot reflect the influence of spectral width. Longuet-Higgins [7] derived a periodic distribution in 1957 under a narrow spectrum assumption. However, some scholars have verified the periodic distribution under the condition that the wave data is a broad spectrum [8,9,10]. The results showed that the theoretical periodic distribution is quite different from the actual wave data, that is to say, this distribution is only applicable to the case where the wave data is a narrow spectrum [11,12]. Meanwhile, the joint distribution of wave height and period is of great significance in practical applications [13]. However, these joint distribution functions of wave heights and periods are derived under the condition of a narrow spectrum, which has certain limitations in the study of ocean waves [14,15].

During the research of ocean wave elements, and based on the maximum entropy principle, researchers have proposed different constraint conditions in accordance with the actual fact of wave height, and presented some maximum entropy distributions of wave height [16]. Some researchers further pointed out that the distributions obeyed by random variables should follow the maximum entropy principle [17]. Namely, the distributions commonly used for hydrologic frequency analysis can be deduced by the maximum entropy principle with different constraint conditions, such as the normal distribution. In view of general applicability of the maximum entropy distribution, it can constrain the existing data while maintaining the greatest uncertainty for the unknown information, so that the priori and artificial nature of the traditional methods can be avoided to a certain extent. Currently, the maximum entropy principle has been extensive applied to many fields [18,19,20].

In this article, work will be done in two aspects. Firstly, a periodic maximum entropy distribution function with four undetermined parameters is derived on the basis of the maximum entropy principle [21,22] and by means of coordinate transformation and solving first variational problems. Secondly, a double entropy joint distribution function of wave height and wave period is derived from the maximum entropy wave height function and the maximum entropy periodic function [23,24], with the help of structures of the Copula function. The new distribution function is not limited by weak nonlinearity, nor by normal stochastic process and narrow spectrum, and it can fit the observed data more carefully [25,26]. It can also be more widely applicable to nonlinear waves in various cases [27,28], owing to the many undetermined parameters it contained. It can thus reflect the uncertainty of wave elements in some cases and can be better applied in theoretical study and practical application.

## 2. Periodic Distribution Function Based on the Maximum Entropy Principle

If the wave period *T* is regarded as a nonnegative continuous random variable with finite value, i.e., 0 < *T* < +∞. The information entropy of *T* is:

(1) $$H\left(T\right)=-\int_{0}^{+\infty }f\left(t\right)\ln f\left(t\right)dt,$$

where in *f*(*t*) is a density function of *T* and it obviously satisfies the constraint condition that:

(2) $$\int_{0}^{+\infty }f\left(t\right)dt=1,$$

and *f*(*t*) is subjected to the following constraints:

(3) $$\int_{0}^{+\infty }f\left(t\right)\ln tdt<+\infty ,$$

(4) $$\int_{0}^{+\infty }t^{\xi }f\left(t\right)dt<+\infty ,$$

where *ξ* is a constant. Equations (3) and (4) are only the description of the generally acknowledged fact, which are not prior designated. Equation (3) constrains that when *t* → 0 or *t* → +∞, *f*(*t*) → 0, which is in accord with the objective fact of the statistical distribution of ocean random variables. In practice, *T* is always a positive value and Equation (4) is also conformed to objective facts. When *ξ* is an integer, Equation (4) can be described as:

(5) $${\bar{T}}^{m}=\int_{0}^{+\infty }t^{\xi }f\left(t\right)dt<+\infty ,\;m=1,2,\ldots ,$$

i.e., all moments of *T* exist.

According to the maximum entropy principle [29,30], our task is to figure out the *f*(*t*) that enables the maximum of *H*(*T*) under Equations (2)–(4). Obviously, it is a conditional variational problem.

Regard Equation (1) as a functional:

(6) $$H\left(T\right)=-\int_{-\infty }^{+\infty }F(t,y)dt,$$

wherein *y* = *f*(*t*), *F*(*t*, *y*) = *y*ln*y*.

Then, the Euler-Lagrange equation determined by the conditional variational problem of Equation (6) can be written as:

(7) $$\frac{\partial }{\partial f}\left[-f\ln\left(f\right)+\lambda \left(f-1\right)+bf\ln\left(t\right)-ct^{\xi }f\right]=0,$$

wherein *f* = *f*(*t*) and *λ*, *b*, *c* and *ξ* are all undetermined constants.

It is obtained from Equation (7) that under the above three constraints, the maximum entropy probabilistic density function of the wave period is:

(8) $$f\left(t\right)=at^{b}e^{-ct^{\xi }},$$

wherein *a* = *e*^*λ*−1^ is an undetermined constant.

The parameters in Equation (8) can be obtained using. ${\bar{T}}_{m}$. (the distribution moments of *T*) in the following equations:

(9) $$\{\begin{array}{c} \frac{\Gamma^{2}\left(\frac{b+2}{\xi }\right)}{\Gamma \left(\frac{b+1}{\xi }\right)\Gamma \left(\frac{b+3}{\xi }\right)}=\frac{T_{1}^{2}}{T_{2}}\\ \frac{\Gamma \left(\frac{b+2}{\xi }\right)\Gamma \left(\frac{b+4}{\xi }\right)}{\Gamma^{2}\left(\frac{b+3}{\xi }\right)}=\frac{T_{1}T_{3}}{T_{2}^{2}}\\ c=\frac{\Gamma^{d}\left(\frac{b+2}{\xi }\right)}{{\left[T_{1}\Gamma \left(\frac{b+1}{\xi }\right)\right]}^{d}}\\ a=\frac{\xi c^{\frac{b+1}{\xi }}}{\Gamma \left(\frac{b+1}{\xi }\right)} \end{array},$$

wherein *T_m_*, *m* = 1, 2, 3, can be obtained from ${\bar{T}}_{m}=\frac{1}{N}\sum_{i=1}^{N}x_{i}^{m},\;m=1,2,3$. *N* represents the number of data points in the dataset. In practice, *x_i_* stands for the *i*-th observation value of *X* and ${\bar{T}}_{m}$ is the estimated value of *T_m_*.

## 3. Double Entropy Joint Distribution Function of Wave Height and Period

The probability density function and the distribution function of wave height can be respectively written as:

(10) $$f\left(H\right)=\alpha H^{\gamma }e^{-\beta H^{n}},$$

(11) $$F_{1}\left(H\right)=\int_{0}^{H}f\left(h\right)dh=\int_{0}^{H}\alpha h^{\gamma }e^{-\beta h^{n}}dh,$$

where the parameters *α*, *γ*, *β*, *n* can be obtained using the method similar to Equation (9).

The distribution function of wave period can be obtained as:

(12) $$F_{2}\left(T\right)=\int_{0}^{T}f\left(t\right)dt=\int_{0}^{T}at^{b}e^{-ct^{d}}dt.$$

The Clayton copula structure function [31,32] is selected when the wave height is positively correlated with the period and the scatter plot has tail correlation in its joint distribution:

(13) $$C\left(u,v\right)={\left(u^{-\theta }+v^{-\theta }-1\right)}^{-1/\theta },$$

where *u* and *v* are corresponding marginal distributions.

The density function *c*(*u*,*v*) obtained from the derivation of the above equation is:

(14) $$c\left(u,v\right)=\frac{\partial C\left(u,v\right)}{\partial u\partial v}=\left(1+\theta \right)u^{-\theta -1}v^{-\theta -1}{\left(u^{-\theta }+v^{-\theta }-1\right)}^{-\frac{1}{\theta }-2}.$$

Presuming that:

(15) $$u=F_{1}\left(H\right)=\int_{0}^{H}\alpha h^{\gamma }e^{-\beta h^{n}}dh,$$

(16) $$v=F_{2}\left(T\right)=\int_{0}^{T}at^{b}e^{-ct^{d}}dt.$$

The double entropy joint distribution function of wave height and period can be obtained by substituting Equations (15) and (16) into Equation (13), which is:

(17) $$F\left(H,T\right)={\left[{\left(\int_{0}^{H}\alpha h^{\gamma }e^{-\beta h^{n}}dh\right)}^{-\theta }+{\left(\int_{0}^{T}at^{b}e^{-ct^{d}}dt\right)}^{-\theta }-1\right]}^{-1/\theta }.$$

The density function *c*(*u*,*v*) obtained by substituting Equations (15) and (16) into Equation (14) is:

(18) $$\begin{array}{cc} c\left(u,v\right) & =\left(1+\theta \right){\left(\int_{0}^{H}\alpha h^{\gamma }e^{-\beta h^{n}}dh\right)}^{-\theta -1}{\left(\int_{0}^{T}at^{b}e^{-ct^{d}}dt\right)}^{-\theta -1}\\ & \cdot {\left[{\left(\int_{0}^{H}\alpha h^{\gamma }e^{-\beta h^{n}}dh\right)}^{-\theta }+{\left(\int_{0}^{T}at^{b}e^{-ct^{d}}dt\right)}^{-\theta }-1\right]}^{-\frac{1}{\theta }-2} \end{array}.$$

The double entropy probability density function of wave height and period can be obtained after substituting Equations (10) and (18) into the following equation, which is:

(19) $$\begin{array}{cc} f\left(x,y\right) & =\frac{\partial F\left(x,y\right)}{\partial x\partial y}=\frac{\partial C\left(F_{X}\left(x\right),F_{Y}\left(Y\right)\right)}{\partial x\partial y}\\ & =\frac{\partial C\left(u,v\right)}{\partial u\partial v}=c\left(u,v\right)f_{X}\left(x\right)f_{Y}\left(Y\right) \end{array}.$$

The double entropy probability density function of wave height and period can be obtained as:

(20) $$\begin{array}{l} f\left(H,T\right)=\left(1+\theta \right){\left(\int_{0}^{H}\alpha h^{\gamma }e^{-\beta h^{n}}dh\right)}^{-\theta -1}{\left(\int_{0}^{T}at^{b}e^{-ct^{d}}dt\right)}^{-\theta -1}\\ \cdot {\left[{\left(\int_{0}^{H}\alpha h^{\gamma }e^{-\beta h^{n}}dh\right)}^{-\theta }+{\left(\int_{0}^{T}at^{b}e^{-ct^{d}}dt\right)}^{-\theta }-1\right]}^{-\frac{1}{\theta }-2}\cdot \alpha H^{\gamma }e^{-\beta H^{n}}\cdot aT^{b}e^{-cT^{d}} \end{array}.$$

The above distribution functions and density functions are not restricted by normal stochastic processes and narrow-spectrum assumptions. The marginal distributions are derived following the maximum entropy principle, so that they can better reflect the uncertainty of ocean waves in a certain physical sense.

## 4. Double Entropy Joint Distribution Function and Engineering Application Thereof

**Figure 1:**
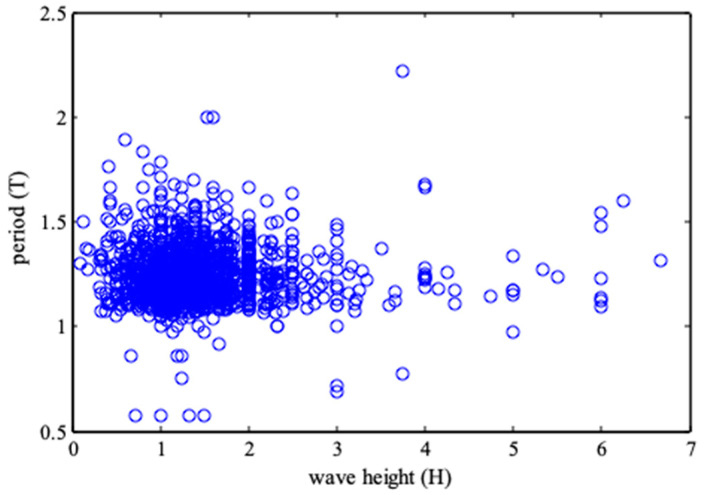
Scatter plot of measured wave height and period.

In this paper, the double entropy joint distribution function is applied to and analyzed through the measured data of mean wave height and mean period by Chaolian Island (1963–1989). Figure 1 is a scatter plot of dimensionless wave heights and periods (“wave height and period” in short).

The marginal distributions of wave heights and periods are selected and adopted as shown in Equations (11) and (12), respectively. The corresponding parameters can be obtained through Equations (7) and (18), as shown in Table 1.

The maximum entropy probability density functions of wave height and period are respectively obtained by substituting the parameters of Table 1 into Equation (19) and are as follows:

(21) $$f\left(H\right)=65.4215H^{-10.2738}e^{-0.1338H^{5.6174}},$$

(22) $$f\left(T\right)=0.9651T^{11.2852}e^{-0.3649T^{6.4199}}.$$

**Figure 2:**
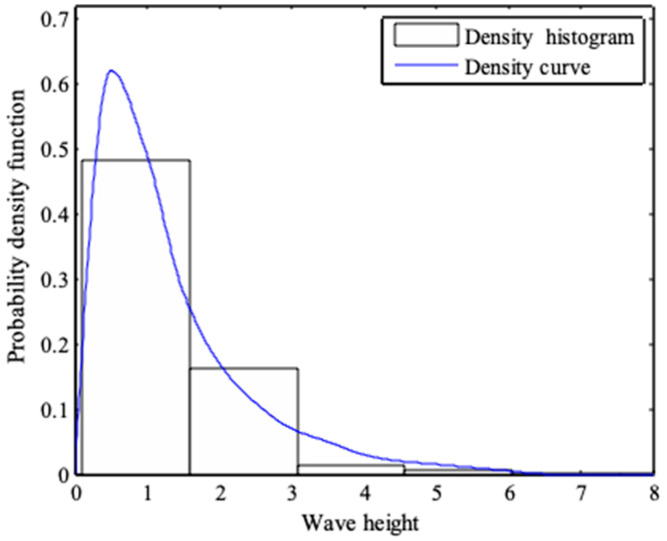
Probability density function of dimensionless wave height.

**Figure 3:**
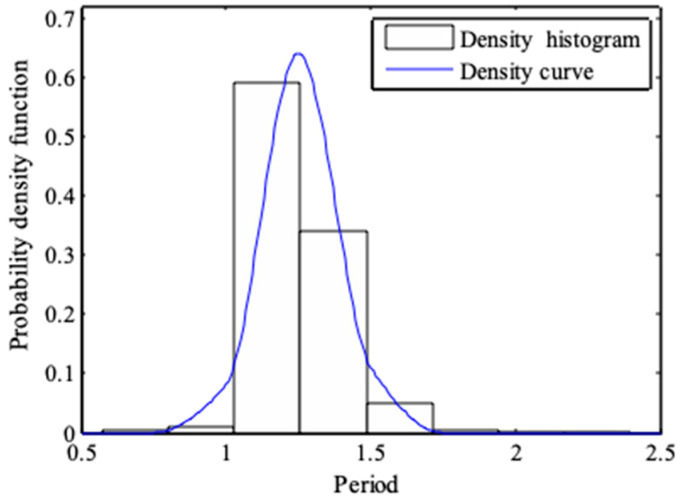
Probability density function of dimensionless wave period.

**Figure 4:**
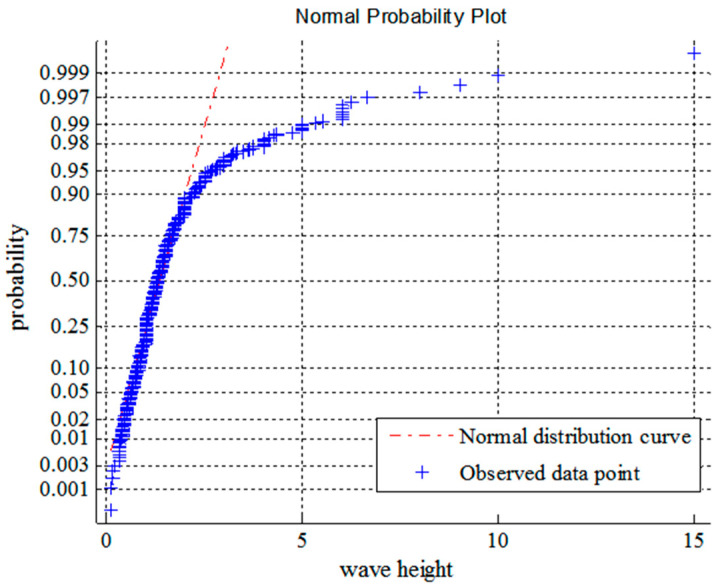
Probability chart for normality test of wave height.

**Figure 5:**
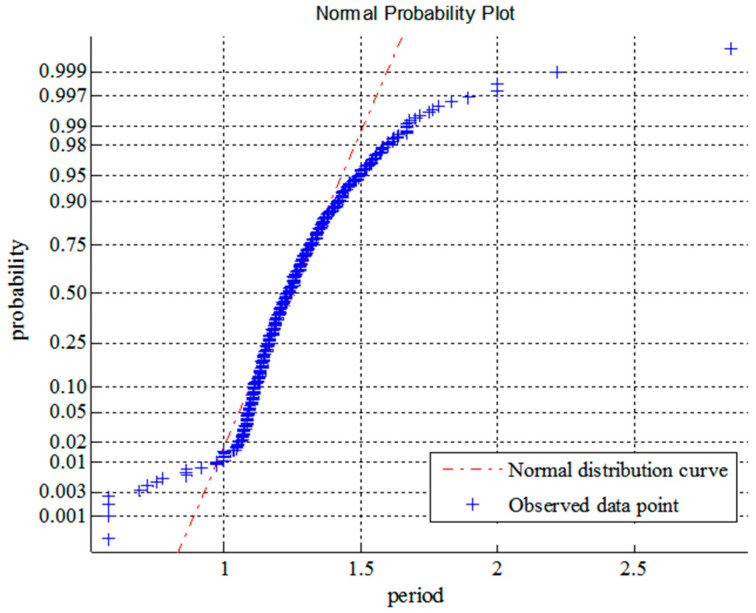
Probability chart for normality test of wave period.

From Figure 2 and Figure 3, it can be seen that the probability density functions of wave heights and periods, which is derived from the maximum entropy principle, are in good agreement with the actual data [33,34]. Figure 4 and Figure 5 show the normality tests for wave height and period, and it can be seen that wave heights and periods do not conform to normal distribution within a large range. However, previous probability density functions are usually derived under the assumption of normal stochastic processes.

The correlation between wave height and period is measured by the Kendall’s tau coefficient *τ* [35], which is calculated by:

(23) $$\tau =\frac{2}{n\left(n-1\right)}\sum_{1\leq i\leq j\leq n}sign\left[\left(x_{i}-x_{j}\right)\left(y_{i}-y_{j}\right)\right].$$

In this equation, (*x_i_*, *y_i_*) is a measured datum, sign (·) is a sign function, when (*x_i_* − *x_j_*) × (*y_i_* − *y_j_*) > 0, sign = 1; when (*x_i_* − *x_j_*) × (*y_i_* − *y_j_*) < 0, sign = −1; when (*x_i_*− *x_j_*) × (*y_i_*− *y_j_*) = 0, sign = 0 and *n* stands for the data series length. According to the measured data, the Kendall’s tau coefficient of wave height and wave is obtained, which is *τ* = 0.0102.

In this paper, the parameters of the Copula function are estimated by the correlation index, that is, the parameter *q* is calculated using the relation between the parameter *q* of the Copula function and Kendall’s tau coefficient *t*. The following equation shows the detailed equation of *q* and *t*.

(24) $$\tau =4\int_{0}^{1}\int_{0}^{1}C\left(u,v\right)dC\left(u,v\right)-1.$$

Taking *τ* = 0.0102 into Equation (24), we can obtain that the Sum of Squares of Deviations of the Clayton Copula function is 0.326, parameter *q* is 2.2056. The Sum of Squares of Deviations (OLS) is calculated by Equation (25).

(25) $$\mathrm{OLS}=\sqrt{\frac{1}{n}\sum_{i=1}^{n}{\left(pe_{i}-p_{i}\right)}^{2}},$$

wherein *pe_i_* is the empirical frequency and *p_i_* stands for the theoretic frequency.

By substituting the parameters of Table 1 and the parameter *q* of the Copula function into Equations (14) and (16), we can obtain the double entropy joint probability density function of wave height and period, which is:

(26) $$\begin{array}{l} f\left(H,T\right)=3.2056{\left(\int_{0}^{H}65.4215h^{-10.2738}e^{-0.1338h^{5.6174}}dh\right)}^{-3.2056}{\left(\int_{0}^{T}0.9651t^{11.2852}e^{-{0.3649t}^{6.4199}}dt\right)}^{-3.2056}\\ \;\cdot {\left[{\left(\int_{0}^{H}65.4215h^{-10.2738}e^{-{0.1338h}^{5.6174}}dh\right)}^{-2.2056}+{\left(\int_{0}^{T}{0.9651t}^{11.2852}e^{-{0.3649t}^{6.4199}}dt\right)}^{-2.2056}-1\right]}^{-2.4534}\\ \;\cdot 65.4215H^{-10.2738}e^{-0.1338H^{5.6174}}\cdot 0.9651T^{11.2852}e^{-0.3649T^{6.4199}} \end{array},$$

And the corresponding distribution function is:

(27) $$F\left(H,T\right)={\left[{\left(\int_{0}^{H}{65.4215h}^{-10.2738}e^{-{0.1338h}^{5.6174}}dh\right)}^{-2.2056}+{\left(\int_{0}^{T}{0.9651t}^{11.2852}e^{-{0.3649t}^{6.4199}}dt\right)}^{-2.2056}-1\right]}^{-0.4534}.$$

**Figure 6:**
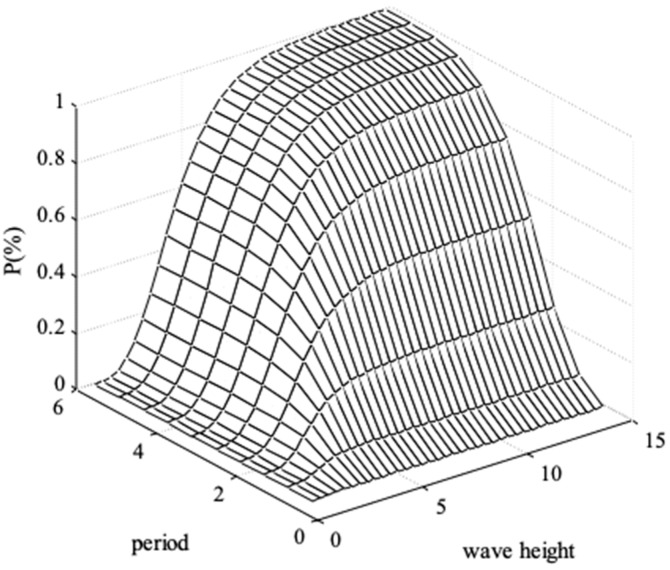
Joint distribution of wave height and period.

**Figure 7:**
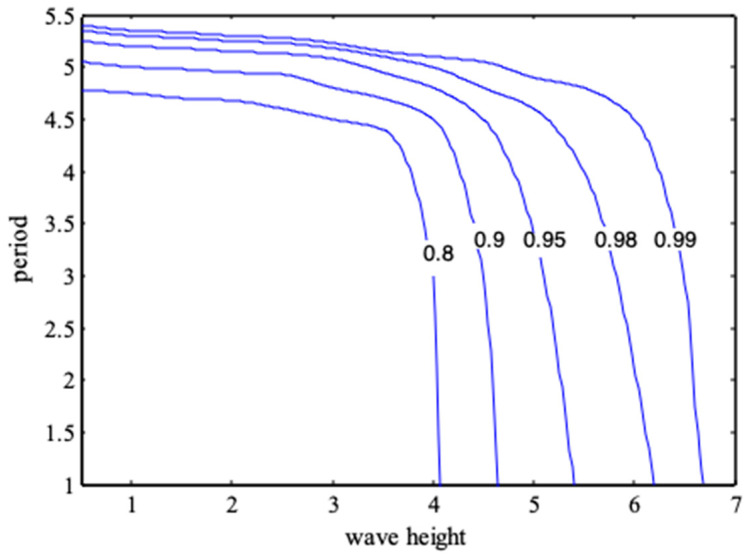
Contour map of joint distribution of wave height and period.

The joint distribution and contour of wave height and period are shown in Figure 6 and Figure 7.

The joint density function of wave height and period is shown as the following equation, which is derived under normal stochastic process and narrow spectrum condition:

(28) $$f\left(\alpha ,\tau \right)=\frac{\pi \alpha^{2}}{4\upsilon \tau^{2}}\left(1+e^{-\frac{\pi \alpha^{2}}{\upsilon^{2}\tau }}\right)\exp\left\{-\frac{\pi \alpha^{2}}{4}\left[1+\frac{1}{\upsilon^{2}}{\left(\frac{1}{\tau }-1\right)}^{2}\right]\right\}.$$

Comparing Equation (28) with the double entropy joint density function, we can obtain the OLS values and parameters of the two joint distributions (see Table 2). The parameter of the joint distribution of wave height and period shown in Equation (28) is *v* = 0.4 and OLS = 0.672.

It can be observed that the sum of squares of deviations between the joint distribution of wave heights and periods based on the maximum entropy of a single variable is relatively small, which shows the superiority of such distributions [36,37].

The recurrence period of wave height and period in engineering is defined as:

(29) $$N_{H}=\frac{1}{1-F\left(H\right)},$$

(30) $$N_{T}=\frac{1}{1-F\left(T\right)},$$

wherein *N_H_* and *N_T_* are the single variable recurrence period of wave height and period respectively. When the period *T* ≥ *t* is satisfied, the conditional probability distribution of wave height *H* is:

(31) $$\begin{array}{cc} F_{H/T} & =P\left(H\leq h|T\geq t\right)=\frac{P\left(T\geq t|H\leq h\right)P\left(H\leq h\right)}{P\left(T\geq t\right)}\\ & =\frac{\left(1-P\left(T\leq t|H\leq h\right)\right)P\left(H\leq h\right)}{P\left(T\geq t\right)}\\ & =\frac{P\left(H\leq h\right)-P\left(T\leq t|H\leq h\right)P\left(H\leq h\right)}{P\left(T\geq t\right)}\\ & =\frac{F\left(H\right)-F\left(H,T\right)}{1-F\left(T\right)}\;(T\geq t) \end{array}.$$

When the wave height *H* ≥ *h* is satisfied, the conditional probability distribution *T* is as follow:

(32) $$\begin{array}{cc} F_{T/H} & =P\left(T\leq t|H\geq h\right)\\ & =\frac{P\left(H\geq h|T\leq t\right)P\left(T\leq t\right)}{P\left(H\geq h\right)}\\ & =\frac{F\left(T\right)-F\left(H,T\right)}{1-F\left(H\right)}\;(H\geq h) \end{array}.$$

**Figure 8:**
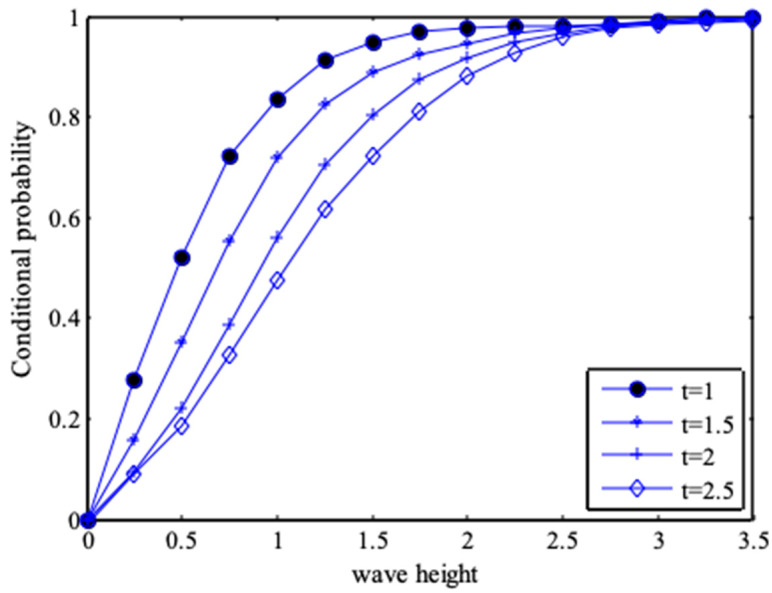
Conditional distribution of wave height under different periods.

**Figure 9:**
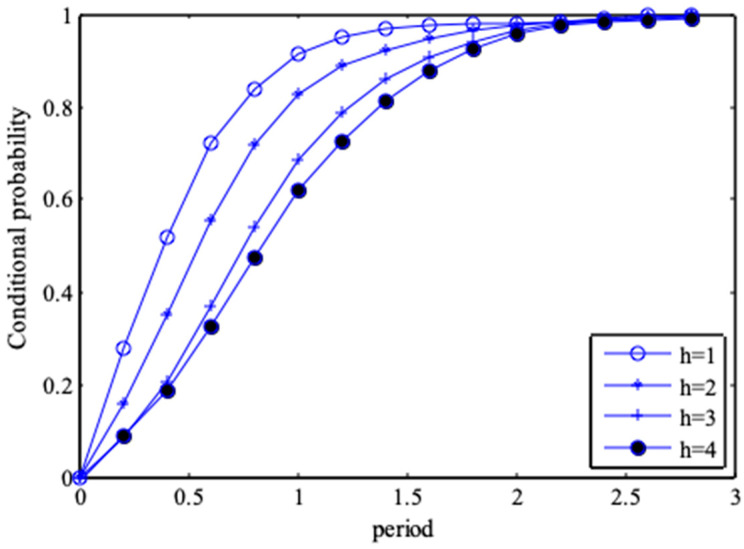
Conditional distribution of periods under different wave heights.

The probability values of wave heights for different periods, and the periodic probability values for different wave heights can be calculated from Equations (31) and (32). Figure 8 and Figure 9 are conditional probability diagrams for different combinations of wave heights and periods. Table 3 shows the probability values of wave heights for different periods, for example, if the period is determined as *T* ≥ 2, *P*(*H* ≤ 2, *T* ≥ 2) = 0.8849.

The joint distribution function of wave height and period is (27). It is recorded that the double entropy joint recurrence period of wave height and period is calculated as follows: *F*(*H*,*T*)

(33) $$N_{H,T}=\frac{1}{1-C\left(F\left(H\right),F\left(T\right)\right)}=\frac{1}{1-F\left(H,T\right)}.$$

The values of wave height and period related to the single variable wave heights, and the period when the recurrence period is 5, 10, 20, 50, 100, 200 and 500 years are obtained by Equations (29) and (30), and the corresponding joint recurrence periods are obtained by Equation (33) (see Table 4).

As can be seen from Table 4, when the wave heights and periods are 4.74 and 2.17 respectively, the single variable recurrence periods of wave heights and periods are 100 years and the combined recurrence period is 50.79 years. That means, the joint recurrence period of wave height and period is less than the recurrence period of its single variable distribution. From the perspective of design value, in the same recurrence period, the design value calculated from the single variable wave height distribution, and single variable periodic distribution are both less than that calculated from the combined distribution of wave height and period.

From the double entropy joint distribution function (Equation (27)) and the traditional joint distribution function (28), the wave heights and periods at the recurrence periods of 5, 10, 20, 50, 100, 200, 500 years can be obtained (see Table 5).

It can be seen from Table 5 that for the same recurrence period, the recurrence level of wave height and period derived from the traditional joint distribution function (28) is lower than that derived from the double entropy joint distribution function in this paper. Obviously, from the perspective of practical ocean engineering design, the traditional method underestimated the combined recurrence level of wave height and period, making its safety reduced.

The above results show that both the design values calculated by the single variable wave height or period and the design values calculated by the traditional joint distribution are relatively small. Therefore, for safety reason, the design values calculated by the new joint distribution function of wave height and period are safer, which can provide a theoretical basis for the design of coastal engineering.

## 5. Conclusions

(1) Based on the maximum entropy principle, a new periodic distribution is deduced in this paper, which loosens the restrictions. The fitting test of the distribution with the observed data shows that the new model deduced in this paper fits the measured data well.

(2) By discussing the structural correlation between the probability distribution modes of wave height and period, and by using the Copula function, a new double entropy joint distribution function of wave height and period is derived. The double entropy distribution function is a non-linear distribution which is derived not from the condition of normal random process and narrow spectrum. The distribution of wave height and period satisfies the maximum entropy principle and reflects the uncertainty of wave elements in a certain physical sense. The joint distribution is verified by the measured data and it is compared with the previous joint distribution of wave height and period. The results show that this distributional function fits well with the measured data and can be more widely used to describe general wave height and period.

(3) Comparing the joint recurrence level calculated by the double entropy joint distribution function, that by the previous joint distribution function of wave height and period and that by the single variable distribution, it is found that both the recurrence level calculated from the previous joint distribution function of wave height and period, and from the single variable distribution are relatively small. For marine engineering applications, the recurrence level obtained from the double entropy joint distribution function of wave height and period is relatively safe.

## Figures and Tables

**Table 1:** Parameter values in distribution functions of wave height and period.

*α*	*β*	*γ*	*n*
65.4215	0.1338	−10.2738	5.6174
***a***	***c***	***b***	***d***
0.9651	0.3649	11.2852	6.4199

**Table 2:** Parameters and OLS values of joint distribution of wave height and period based on maximum entropy of a single variable.

*α*	*β*	*γ*	*n*	*θ*	OLS
65.4215	0.1338	−10.2738	5.6174	2.2056	0.326
***a***	***c***	***b***	***d***		
0.965	0.3649	11.2852	6.4199		

Note: OLS represents the criterion of the minimum of sum square variation.

**Table 3:** Probability of wave height under different period values.

Height/m Probability Period/s	1	1.5	2	2.5
1	0.8612	0.9508	0.9824	0.9981
1.5	0.7623	0.9121	0.9638	0.9976
2	0.5613	0.8499	0.9487	0.9884
2.5	0.5027	0.7973	0.9223	0.9614

**Table 4:** Recurrence period of the single variable distribution and the corresponding recurrence period of joint distribution.

Recurrence Period of Single Variable	Joint Recurrence Period	Marginal Distribution Design Value	
*N*	*N*_*H*,*T*_	Wave Height/m	Wave Period/s
5	2.88	1.26	1.18
10	5.37	1.95	1.25
20	10.26	2.55	1.42
50	25.58	3.25	1.58
100	50.79	4.74	2.17
200	100.81	5.19	2.40
500	250.29	5.76	2.53

**Table 5:** Joint recurrence level of the wave heights and periods of the two distribution functions.

Once upon *N* Years	Double Entropy Joint Distribution		Traditional Joint Distribution (Equation (30))	
	Wave Period/s	Wave Height/m	Wave Period/s	Wave Height/m
5	1.18	1.48	1.02	1.36
10	1.25	2.15	1.13	2.05
20	1.42	2.76	1.22	2.64
50	1.58	3.41	1.38	3.31
100	2.17	4.95	1.87	4.79
200	2.40	5.51	2.09	5.20
500	2.53	6.07	2.35	5.86
